# 

**Published:** 1879-02

**Authors:** 


### Books and Pamphlets Received.

An Atlas of Human Anatomy. Illustrating most of the Ordinary Dissections, and many not usually practiced by the Student. Accompanied by an Explanatory Text. By Rickman John Godlee, M. S., F. R. C. S. Philadelphia: Lindsay & Blakiston. Part I. Price, $2.50.

Modern Medical Therapeutics; A compendium of recent Formulæ and Specific Therapeutical Directions. From the Practice of Eminent Contemporary Physicians, American and Foreign. By George H. Napheys, A. M., M. D., etc. Sixth Edition. Enlarged and Revised. Philadelphia: D. G. Brinton, 115 South Seventh Street. 1879. pp. 590.

A Practical Treatise on the Diseases of the Testes, and of the Spermatic Cord, and Scrotum. By T. B. Curling, F. R. S., Consulting Surgeon to the London Hospital, etc. Fourth Edition. Revised and Enlarged. Philadelphia: Lindsay & Blakiston. 1878. pp. 645.

Physiology; Preliminary Course Lectures. By James T. Whittaker, M. A., M. D. On the Influence of Physiology upon Practice; On the Conservation of Forces; On the Origin of Life, and the evolution of its forms; and on Protoplasms, Bone, Muscle, Nerve and Blood. Illustrated. Cincinnati: Chauncy R. Murry, 103 W. Sixth St. 1879. pp. 272.

Conspectus of Organic Materia Medica and Practical Botany, Comprising the Vegetable and Animal Drugs; Their Physical Character, Geographical Origin, Classification, Constituents, Doses, Adulterations, &c. By L. E. Sayre, Ph. G. Philadelphia: D. G Brinton, 115 South Seventh St. 1879. pp. 211.

Differential Diagnosis; A Manual of the Comparative Semeiology of the more important Diseases. By F. de Havilland Hall, M. D., Assistant Physician to the Westminster Hospital, London. Philadelphia: D. G. Brinton, 115 South Seventh Street. 1879. pp. 201.

A Practical Manual of the Diseases of Children with a Formulary. By Edward Ellis, M. D. New York: Wm. Wood & Co., 27 Great Jones Street. 1879. pp. 210.

Report upon the case of the late Dr. E. A. Groux, to the Brooklyn Anatomical and Surgical Club. By Charles Jewett, M. D.

Fifty Years Ago. An Address to the Graduating Class of the Medical College of the Pacific, for 1878. By Henry Gibbons, Sr., M. D., Prof. of the Principles and Practice of Medicine and Clinical Medicine.

Annual Report of the Rhode Island Hospital. 1878.

Consumption and its Treatment, with the Hypophosphites. By J. A. McArthur, M. D. (Harv.)

Proceedings of the Board of Experts, Authorized by Congress, to investigate the Yellow Fever Epidemic of 1878. Meeting held in Memphis, Tenn., Dec. 26th to 28th, 1878.

Transactions of the American Opthalmological Society. Twelfth, Thirteenth and Fourteenth Annual Meetings. 1878.

Twenty-Sixth Annual Announcement of the Medical Department of the University of Vermont, for the year 1879.

Optic Neuritis, with notes of three Cases. By C. J. Lundy, M. D.

Vick's Floral Guide. No. 1. 1879.

On the Fracture of the Femur. By Edward Borck, M. D. With Illustrations. St. Louis, Mo.

Twenty-sixth Annual Report of the Pennsylvania Training School for Feeble-minded Children. Media, Delaware Co., Pa.

F8 (4c9)

THE BUFFALO

### MEDICAL AND SURGICAL

Books and Pamphlets Received,.... 280

THE BUFFALO

MEDICAL AND SURGICAL JOURNAL.

PUBLISHED MONTHLY,—Containing Original Articles, Reports of Medical Societies and Hospitals, Editorial, Reviews, Correspondence, Army News, etc., etc; including the usual variety of Medical Periodical Publications. Specimen copies sent on application to BUFFALO MEDICAL AND SURGICAL JOURNAL, J. F. MINER, M. D., 978 Main Street, BUFFALO, N. Y.

TERMS OF SUBSCRIPTION, $3 00 PER YEAR, IN ADVANCE,—All communications for publication should be sent before the fifteenth (15) of each month, or they will of necessity lie over until the next number. No change can be made in advertisements after the twenty-fifth.

Correspondence and original articles are solicited. Publication is no evidence that the views of the author are endorsed by the Journal.

